# Mapping Gilles de la Tourette syndrome through the distress and relief associated with tic-related behaviors: an fMRI study

**DOI:** 10.1038/s41398-023-02711-z

**Published:** 2024-01-08

**Authors:** Laura Zapparoli, Francantonio Devoto, Marika Mariano, Silvia Seghezzi, Domenico Servello, Mauro Porta, Eraldo Paulesu

**Affiliations:** 1 Psychology Department and NeuroMi – Milan Centre for Neuroscience, University of Milano-Bicocca, Milan, Italy; 2 fMRI Unit, IRCCS Orthopedic Institute Galeazzi, Milan, Italy; 3 Institute of Cognitive Neuroscience, University College London, London, UK; 4 Tourette Center, IRCCS Orthopedic Institute Galeazzi, Milan, Italy

**Keywords:** Neuroscience, Psychiatric disorders

## Abstract

Personal distress associated with tic urges or inhibition and relief associated with tic production are defining features of the personal experience in Gilles de la Tourette syndrome (GTS). These affective phenomena have not been studied using fMRI, hindering our understanding of GTS pathophysiology and possible treatments. Here, we present a novel cross-sectional fMRI study designed to map tic-related phenomenology using distress and relief as predicting variables. We adopted a mental imagery approach and dissected the brain activity associated with different phases of tic behaviors, premonitory urges, and the ensuing tic execution or inhibition: these were compared with the mental simulation of “relaxed situations” and pre-determined stereotyped motor behaviors. We then explored whether the ensuing brain patterns correlated with the distress or relief perceived for the different phases of the tasks. Patients experienced a higher level of distress during the imagery of tic-triggering scenarios and no relief during tic inhibition. On the other hand, patients experienced significant relief during tic imagery. Distress during tic-triggering scenarios and relief during tic imagery were significantly correlated. The distress perceived during urges correlated with increased activation in cortical sensorimotor areas, suggesting a motor alarm. Conversely, relief during tic execution was positively associated with the activity of a subcortical network. The activity of the putamen was associated with both distress during urges and relief during tic execution. These findings highlight the importance of assessing the affective component of tic-related phenomenology. Subcortical structures may be causally involved in the affective component of tic pathophysiology, with the putamen playing a central role in both tic urge and generation. We believe that our results can be readily translated into clinical practice for the development of personalized treatment plans tailored to each patient’s unique needs.

## Introduction

Gilles de la Tourette syndrome (GTS) is a neurological movement disorder characterized by motor and sound tics lasting at least 12 months [1]. Tics are movements or sounds that appear “repetitive, seemingly uncontrollable, out of context, and exaggerated” [2]. Tics are usually preceded by a *premonitory urge*, typically described as a “mounting internal tension”, which can be temporarily relieved only by tic expression [3, 4]. These motoric manifestations still lack a mechanistic explanation: the main proposal is that tics should be associated with aberrant activity in the basal ganglia and the cortico-striato-thalamo-cortical circuits [5–8].

GTS is associated with psychopathological co-morbidities [1, 9, 10], but tics and premonitory urges remain the core features of GTS. Tics can be controlled for a certain time through voluntary tic suppression, something that differentiates GTS from other movement disorders, such as Parkinson’s disease or Huntington’s disease [11, 12]. Furthermore, tics are not fully voluntary nor fully automatic, but they are said to be “unvoluntary” [3], to indicate their quasi-voluntary nature.

Tic suppression, typically more urgent in social contexts [13], comes at the cost of considerable personal distress that can be relieved only once tics are left free to express. These affective phenomena have never been investigated with fMRI. Yet, capturing their distinctive attributes may help differentiate the physiology of tics from that of ordinary actions. However, the task of studying GTS tic behavior and the ensuing subjective perceived sensation with fMRI is hampered by the artifacts related to head movements: the comparison with tic suppression is unreliable because the movement artifacts hamper only one condition.

### The neurofunctional correlates of tics and their premonitory urges

Investigating the anatomical substrate of tics has proven challenging because of the movement artifacts. One outstanding question concerns the anatomical origin of tics and their suppression, and how these compare physiologically with voluntary actions or their inhibition [14, 15]. Most of the available task-based studies were based on small samples of patients, a matter of concern in terms of statistical power and control of false positives [16, 17].

For example, Bohlhalter et al. [18] and Neuner et al. [19] investigated the neurofunctional correlates of tics in small samples of GTS patients. They characterized the BOLD response before tic onset and during tic manifestation. Before tics, the authors reported activation of a cortical and subcortical network, including SMA, the parietal operculum, the anterior cingulate and the insular cortex. At the onset of tics, increased activity was reported in the primary motor cortex and the cerebellum [18, 19].

Crucially, none of these studies discriminated between the fMRI patterns of actual tics and voluntary movements. Addressing this limitation, Wang et al. [20] provided evidence that actual tics, when contrasted to imitated tic-like movements collected in healthy controls, are associated with augmented brain activity at the level of sensorimotor pathways within the cortico-basal ganglia circuits. The authors suggested that this activity may represent a “feature of premonitory urges that generate spontaneous tic behaviors” [20]. However, none of the mentioned experimentts investigated the different phases of tic behavior using an appropriate matched control condition in the same sample of patients.

Clearly, urges and tics or their inhibition come lumped together, and it remains questionable whether the fMRI’s temporal resolution is sufficient to separate these phenomena. Furthermore, tics are hardly comparable with voluntary movements and are likely to generate artifacts in the fMRI data; urges are perhaps more treatable, yet it remains to be decided whether they represent an anxiety-like symptom and whether the higher pre-recruitment of motor/premotor circuits can be differentiated from similar patterns seen for typical “ready, set, go tasks” as described in humans and in monkeys; finally, one can still argue on whether tic inhibition is fully comparable with a no-go task in normal controls or GTS patients themselves. One key to disentangling some of these outstanding issues would be having a measurable variable typically associated with the tic phenomenology but not the control behaviors of choice. Distress associated with urges or tic inhibition and relief associated with tic production could represent suitable variables.

### Aims of the study

Here we present a novel experimental paradigm to study the neurofunctional correlates of tic manifestations in GTS patients and the brain correlates of personal distress or relief. To avoid the confounds introduced by unwanted patients’ movement, we used a mental imagery task of the behaviors associated with tic manifestation, inhibition, and urges.

To make the tic imagery activity better connected to patients’ actual experience, we asked them to recall typical tic-provoking situations, see [13]. We compared these fMRI patterns with the neural activity induced by the mental simulation of “relaxed situations” and pre-determined stereotyped motor behaviors. We also evaluated the distress or relief perceived during the different phases of the task, using trial-by-trial subjective reports, and we correlated these with brain activity.

### Strengths of our experimental approach

First, the adoption of a mental imagery task would allow engaging with the different phases of tic behaviors (premonitory urges, followed by tic execution or tic inhibition) without motion artifacts. Crucially, the formal comparison with a matched baseline condition allowed us to “subtract” the neural activity generally associated with motor behavior not directly related to the tic dimension. Moreover, the presence of subjective reports allows us to assess whether this mental simulation was effective in evoking tic-related scenarios (e.g., a greater level of distress perceived during the imagery of tic-related contexts). Finally, specific correlations between the brain activity recorded during the mental simulations of the different phases and the level of self-reported distress might be considered as proof that the evoked brain patterns are specifically related to the disease dimension.

### Expected results

We anticipated a greater level of distress during the tic-evoking scenarios than the retrieval of neutral contexts. One such finding would provide a validation of our experimental design.

At the neuroanatomical level, one could anticipate that any relief due to tic imagination might be associated with the activity related to reward processing: one such finding would point to tics as pathologically motivated behaviors enacted to avoid a negative affective state. Conversely, the relief might be associated with augmented activity within the motor system itself, leaving the question of whether a positive correlation could be found in cortical motor areas or subcortical motor structures. Similar considerations could be made with the individual distress associated with premonitory urges: we expected this to be correlated with brain regions previously associated with anxiety and mood disorders, the question being whether the higher activity could also be seen in motoric regions, pointing to premonitory urges as sensorimotor phenomena and to the enhanced response being associated, perhaps, to a pre-motoric pre-alarm. Finally, as distress during urges and relief following tic expression could be seen as the opposite sides of the same coin, we anticipated that some brain regions might show positive correlations with these two sides for different moments of our paradigms: distress during imagery evoked urges and relief following tic imagery expression. If these could be identified, they should be strong candidates for being causally related to the generation of tics and their phenomenology.

## Material and methods

### Participants

We carried out an a-priori power analysis on the basis of the scientific literature (see supplementary materials). Twenty-five GTS patients (GTS, age: 26.3 ± 6.4 years; education: 12.26 ± 3.4 years; male/female: 20/5) participated in this study. All the participants were right-handed [21]. The study protocol was approved by the local Ethics Committee (Prot. SOA, 149/INT/2016), and informed written consent was obtained from all subjects according to the Helsinki Declaration (1964). All participants completed a neuropsychological and psychopathological assessment and a detailed interview about the severity of their symptoms (see supplementary materials). The details of the clinical and neuropsychological data are reported in Table 1 and in Supplementary Table S1. Most patients (*n* = 19) were on medication with neuroleptics. Molecules and dosages are reported in Table 1.

**Table 1:** Demographical, neuropsychological, and clinical data collected at the moment of the experiment.

#	*Sex*	*Age*	*Edu*	*MMSE*		*FAB*		*Raven*		*BIS-11*	*YBOCS*	*BDI*	*ASRS*	*STAI-X-1*	*STAI-X-2*	*PUTS*	*YGTSS*			*Pharmacological treatment*
				*Raw*	*Corr*	*Raw*	*Corr*	*Raw*	*Corr*								*Moto*	*Fon*	*Social*	
1	M	23	8	29	28.19	18	18	31	30.5	65	24	13	2	31	40	21	13	11	20	Risperidone
2	F	19	12	24	22.59	16	14.9	18	16	77	19	22	2	62	56	26	21	21	50	None
3	M	22	13	30	30	18	18	35	32.5	58	15	12	2	41	50	29	15	5	20	None
4	M	20	12	30	30	17	15.9	28	26	62	2	10	0	55	53	34	20	17	20	Aripiprazole (22.5 mg); Pimozide (4 mg)
5	M	23	13	30	30	18	18	35	32.5	58	13	1	2	31	54	24	17	7	30	Aripiprazole (7.5 mg); Pimozide (2 mg)
6	M	20	13	30	30	18	18	35	32.5	68	16	2	2	45	43	25	11	5	40	Aripiprazole (15 mg)
7	M	25	13	30	30	17	15.6	34	31.5	84	13	7	3	27	43	21	20	14	20	Pimozide (2 mg)
8	M	19	13	26	24.59	16	14.5	35	32.5	71	16	8	4	26	44	32	13	5	0	Quetiapine (50 mg)
9	M	29	10	29	27.59	13	12.1	30	29	55	19	7	4	34	32	28	6	7	20	Pimozide (4 mg)
10	F	21	13	29	27.59	15	13.5	22	19.5	86	16	5	5	47	36	26	12	11	0	Haloperidol (2 mg); Aripiprazole (15 mg)
11	M	29	8	28	27.19	16	15.5	24	24	63	13	4	1	31	45	19	11	10	30	Aripiprazole (30 mg); Pimozide (2 mg)
12	M	27	16	30	30	18	18	36	36	57	20	13	0	41	51	27	14	0	20	Aripiprazole (30 mg)
13	M	19	12	27	25.59	16	14.5	30	28	48	17	2	0	53	40	25	12	13	20	Aripiprazole (15 mg)
14	M	50	8	29	28.97	14	13.9	22	23.5	67	11	0	2	30	25	27	8	7	40	Aripiprazole (30 mg)
15	M	22	13	30	30	18	18	35	32.5	61	20	5	4	37	45	36	15	6	20	Pimozide (4 mg)
16	M	28	16	29	27.59	18	18	30	26.25	70	18	17	1	35	47	34	16	6	40	Aripiprazole (15 mg)
17	M	18	8	30	30	16	15.3	32	32	76	20	7	4	29	33	21	14	0	20	Aripiprazole (22.5 mg); Pimozide (4 mg)
18	M	22	8	30	30	18	18	32	32	70	17	11	4	33	50	10	0	0	50	Aripiprazole (15 mg)
19	M	19	13	30	30	17	15.5	36	36	74	10	7	4	34	52	28	17	0	20	Pimozide (4 mg)
20	M	18	8	29	28.19	16	15.3	34	34	60	0	0	0	24	23	14	3	4	0	Aripiprazole
21	F	34	20	30	30	17	15.3	28	24.5	86	12	11	4	39	50	29	15	12	30	None
22	M	48	13	29	27.89	18	18	30	29.5	68	18	8	2	41	39	36	17	18	20	None
23	M	47	8	27	26.62	16	15.8	28	26.75	67	10	0	1	37	39	22	11	3	0	Pimozide (4 mg)
24	F	24	16	30	30	18	18	34	30	70	19	20	4	59	41	34	16	11	30	None
25	F	32	18	30	30	18	18	29	25.25	51	21	14	2	50	69	33	11	13	40	None

### Experimental task

Participants underwent fMRI scanning while performing a *Guided Mental Imagery* task aimed at mimicking the real-life scenarios of being in a distressful situation and managing the ensuing ticking manifestation. Subjects were visually instructed to engage in mental imagery by following brief instructions appearing on the screen:

- During the ***urge phase***, participants were instructed to imagine being in a distressful situation that promotes tics occurrence (*tic* scenario) or to imagine being in a relaxing situation that does not promote tics insurgence (*control* scenario).
- During the subsequent ***behavior phase***, subjects could be instructed either to imagine **acting the tic** (*tic act scenario*) or a **pre-determined voluntary movement** (*control act scenario*) or to imagine **inhibiting the tic** (*tic inhibition scenario*) or *inhibiting* the voluntary movement (*control inhibition scenario*).
- During a ***rating phase***, subjects were asked to rate the *distress* perceived during the *context* phase (“How much distressful was the imagination of the first situation?”), and the *relief* perceived following the *behavior* phase (“How much relieving was the imagination of the second situation?”) on a five-point Likert scale ranging from 1 (“Not at all”) to 5 (“Extremely”).
- Finally, during the *wash-out* phase, participants were instructed to relax.

**Fig. 1:**
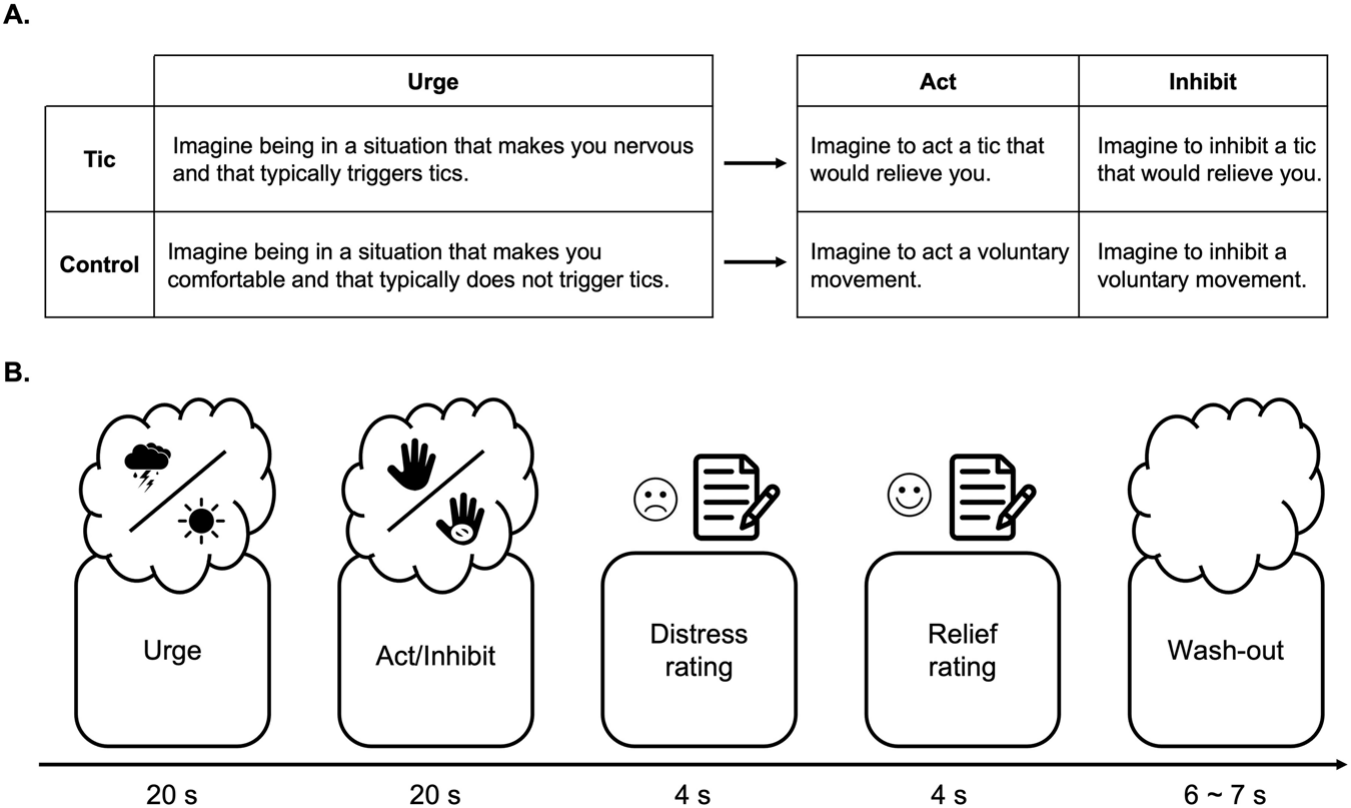
The Guided Mental Imagery task. **A** Instructions given to participants. The instructions given to the participants are reported for each condition. **B** Timing of an example trial. Graphical representation of the Guided Mental Imagery task during fMRI. After tic-triggering scenarios, patients were instructed to imagine their tics (tic imagery execution) or to imagine inhibiting their tics (tic imagery inhibition).

The instructions given to participants and the overall structure of the experimental design are shown in Fig. 1A. The task was repeated 12 times, the order of tic/control blocks was counterbalanced across the subjects; the order of the act/inhibit behavior blocks was randomized within-condition and participants. An example of task structure and timing is given in Fig. 1B.

At the end of the fMRI task, participants rated the *quality of their imagery during the two conditions* by means of two VAS. Finally, they filled in the *Movement Imagery Questionnaire* [22].

### Statistical analyses of the behavioral data

Ratings of the *distress/relief* during the different phases of the task and the *quality of the imagery* assessed post-scan were analyzed by means of paired samples T-test to test the difference between the tic and the control conditions.

We also tested the correlation of the behavioral ratings in the different task phases by calculating Person’s correlation coefficient. This analysis was aimed to justify testing the hypothesis that some brain regions could correlate with more than one behavioral index in different moments of the trials (e.g., during imagination of tic-triggering scenarios and tic inhibition).

Finally, we calculated whether behavioral ratings correlated with the severity of the disease, as assessed by the YGTSS score.

Unless otherwise specified, behavioral data were normally distributed and parametric statistics were employed.

### Statistical analyses of functional magnetic resonance imaging data

All the details about the fMRI data acquisition, data preprocessing, and analysis of head motion parameters are described in the supplementary materials.

#### First-level fixed-effect analyses

Two patients were excluded from the analyses due to technical problems, leading to a final sample of 23 GTS patients. We characterized the brain activity recorded during each task phase (*urge, behavior, rating, and wash-out* phase, more details are reported in the supplementary materials). We included one regressor for each scenario (tic and control) and each phase (urge, act behavior, inhibit behavior, wash-out) and one regressor for the *rating* phase. Different sets of first-level contrasts were performed to compute separate random-effect second-level analyses (see supplementary materials), addressing two different research questions: (i) were the patients performing the guided imagery task? (ii) were there specific neural patterns associated with the different scenarios (tic vs control) and the different phases (urge phase, act phase, inhibit phase)?

(i) **Identification of the main effects of mental motor imagery**. For the terminology used please refer to Fig. 1. We generated, for each participant and each scenario (tic/control), a contrast image of the comparison *urge phase > wash-out phase* and a contrast image of the comparison *behavior phase (act or inhibit) > wash-out*, for a total of six contrast images per subject overall (urge tic, urge control, behavior act tic, behavior act control, behavior inhibit tic, behavior inhibit control).
(ii) **Characterization of urge / task-specific activation patterns**. We generated a contrast image for each participant for the different comparisons, for a total of three contrast images for each subject overall.

**Comparison ii-A** *: Urge phase tic scenario > Urge phase control scenario.*

**Comparison ii-B:** *Behavior phase tic scenario* (***act***) > *behavior phase control scenario (****act****)*.

**Comparison ii-C:** *Behavior phase, tic scenario* (***inhibit***) > *behavior phase control scenario (****inhibit****)*.

#### Second-level random-effect analyses

(i) Identification of the main effects of mental motor imagery
  The contrast images generated in the first-level analyses were entered into two separate second-level random-effect analyses. This analysis conforms to a full factorial analysis (Factor 1: Scenario (tic vs. control), Factor 2: Phase (urge vs. act vs. inhibit)).
(ii) Characterization of task-specific activation patterns
  In our second analysis, we performed three paired-sample t-tests, one for each task phase (urge phase, act phase, inhibit phase). For each analysis, we inserted a behavioral covariate, indicating the level of distress (urge phase) or relief (act and inhibit phases) perceived during the execution of the task.
(iii) Conjunctions of correlations for distress and relief.

The conjunction of the fMRI response for the **distress** effect during urges and **relief** for tic imagery. We tested the hypothesis that the behavioral covariates (e.g., distress during tic-causing scenarios or the relief during tic imagery) could predict the activity of the same brain regions with a similar magnitude of response. These tests were limited to the pairs of variables showing a significant correlation regarding the behavioral measures, namely the **distress** effect during urges and **relief** for tic imagery.

All the results reported survive a correction for multiple comparisons: we used the nested-taxonomy strategy recommended by [23], including regional effects meeting either a cluster-wise or voxel-wise family-wise error rate (FWER) correction. The voxel-wise threshold applied to the statistical maps before the cluster-wise correction was *p* < 0.001 uncorrected, as recommended by [24]. For clusters significant at the *p* < 0.05 FWER-corrected level, we also report the other peaks at *p* < 0.001.

#### Correlations with demographic and clinical data

See supplementary materials.

#### Correlations with neuroleptic medication levels

See supplementary materials.

## Results

### Behavioral results and relationship with clinical data

During the *urge* phase, subjects reported higher *perceived distress* in the tic compared with the control scenario (Student’s t(22) = 4.51, *p* < 0.001). During the *behavior act* phase, subjects reported higher *perceived relief* in the tic compared with the control condition (Student’s t(22) = 2.18, *p* = 0.04), whereas during the *behavior inhibit* scenario they did not report different ratings (Student’s t(21) = −0.09, *p* = 0.93).

The distress rating recorded during the tic-recalling phase (urge phase) was significantly correlated with the relief perceived during tic imagination execution (Person’s *r*(22) = 0.47, *p* = 0.02) but not during tic inhibition (Person’s *r*(22) = −0.23, *p* = 0.29). The distress-relief association was absent for the behavioral rating collected during the imagination of pre-determined movements (Person’s *r*(22) = 0.21, *p* = 0.34) or inhibition of the same movements (Person’s *r*(22) = 0.13, *p* = 0.56).

We further explored the relationship between emotional indicators (distress and relief) and symptom severity assessed by the YGTSS total score: we identified a significant positive correlation between YGTSS scores and the relief perceived during tic imagery (Spearman’s rho(22) = 0.41, *p* = 0.05): patients with more severe symptoms were those who perceived greater relief after tic imagery. The relationship between YGTSS and the distress/relief perceived during urge imagery and tic inhibition were not significant (Spearman’s rho(22) = 0.20, *p* = 0.36; Spearman’s rho(22) = 0.19, *p* = 0.38).

**Fig. 2:**
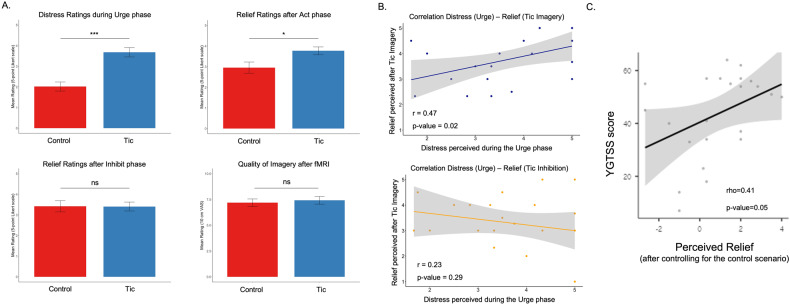
Behavioral results. **A** Self-report distress and relief perceived during the different phases of the experimental and control tasks, and self-report the quality of the imagery for the experimental and control tasks. **B** Correlations between the behavioral measures collected during the different phases of the experimental task. **C** Correlation between the relief perceived during tic imagery and the severity of the disease, as assessed by the YGTSS score.

After fMRI, the average report of the *quality of the imagery* between the experimental tic task and the control task was similar (Student’s *t*(22) = 0.46, *p* = 0.65), indicating similar imagery abilities in both scenarios. See Fig. 2 (descriptive statistics are reported in supplementary materials).

### fMRI Results

#### Main effect of motor imagery and of the specific phases of the task

These results are described in the supplementary materials (Fig. S1 and Table S2).

#### Correlation between level of distress and BOLD signal during the recall of tic-inducing scenarios

**Fig. 3:**
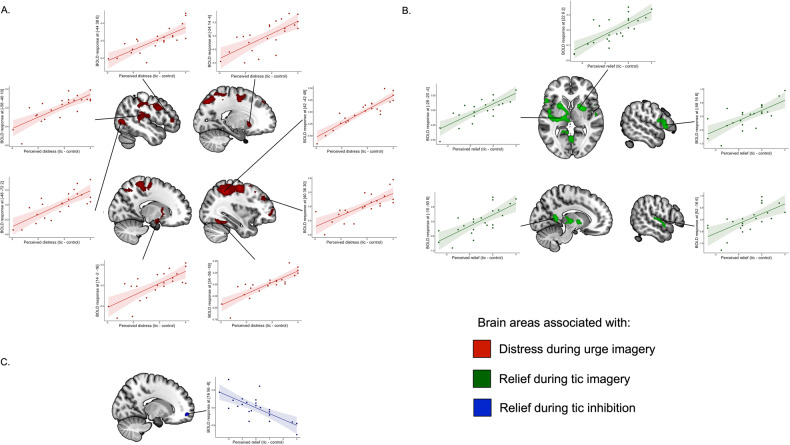
Results of regression analyses. **A** Effect of distress during urge imagery (red). **B** Effect of relief during tic imagery (green). **C** Effect of relief during tic inhibition imagery (blue).

During urge phase of the tic scenario, the activity of a cortico-subcortical network was positively associated with the perceived distress (Table 2A and Fig. 3A). This network included prefrontal the superior medial prefrontal cortex and SMA, the middle and inferior frontal gyri, the middle cingulum, bilaterally, the left precentral and postcentral gyri, the left temporo-occipital region, the right fusiform gyrus. We also found clusters of significant activation in the right caudate nucleus and nucleus accumbens, the putamen, bilaterally, and the right cerebellum.

**Table 2:** fMRI results of the regression analyses.

	Left hemisphere				Right hemisphere				
Anatomical label (Brodmann area)	x	y	z	Z-value	x	y	z	Z-value	Cluster size (voxels)
**A. Effect of distress during context imagery**									
Inferior parietal lobule (BA 40)					42	−42	48	5.91*	9266
					30	−40	50	5.44*	
					50	−38	46	5*	
Superior frontal gyrus (BA 6)					28	−12	60	4.91*	
Superior medial frontal gyrus (BA 8)					8	38	50	4.35	
Medial frontal gyrus (BA 6)	−24	−8	48	4.34					
Supplementary motor area (BA 6)					2	−6	56	4.33	
Precentral gyrus (BA 6)					30	−14	56	4.99*	
Postcentral gyrus (BA 3)	−36	−20	46	4.62					
	−36	−26	46	4.58					
					20	−34	62	4.73*	
Superior parietal lobule (BA 7)	−20	−76	46	4.78*					
	−20	−72	48	4.67#					
	−28	−60	50	4.48					
Supramarginal gyrus (BA 40)					52	−34	46	4.74*	
Inferior parietal lobule (BA 7)					28	−54	50	4.66#	
Fusiform gyrus (BA 37)					34	−56	−18	5.04*	478
					28	−44	−18	4.28	
Cerebellum VI					28	−50	−22	4.44	
					28	−66	−26	3.69	
Middle temporal gyrus (BA 42)	−58	−40	10	4.47					1200
	−42	−42	4	3.25					
	−44	−40	0	3.37					
Superior temporal gyrus (BA 41)	−58	−20	8	4.24					
	−38	−32	8	4					
	−58	−28	8	3.87					
	−38	−36	6	3.47					
Postcentral gyrus (BA 3)	−54	−16	20	3.24					
Supramarginal gyrus (BA 40)	−46	−32	28	3.14					
Rolandic operculum	−42	−30	16	4.18					
	−42	−26	20	4					
	−48	−12	14	3.55					
	−42	−10	14	3.41					
	−42	−18	18	3.33					
	−40	−10	18	3.25					
Middle occipital gyrus (BA 37)	−48	−72	2	4.44					437
	−40	−70	8	3.78					
Inferior occipital gyrus (BA 19)	−36	−72	−6	3.58					
	−40	−70	−6	3.42					
Middle temporal gyrus (BA 42)	−56	−62	6	4.13					
	−42	−66	8	3.61					
	−52	−56	12	3.39					
Inferior temporal gyrus (BA 37)	−50	−56	−6	3.32					
	−46	−58	−6	3.27					
	−46	−64	−6	3.22					
Inferior frontal gyrus, pars triangularis (BA 45)	−44	38	6	4.43					905
Inferior frontal gyrus, pars orbitalis (BA 47)	−34	32	−4	3.31					
Superior medial frontal gyrus (BA 8)	−8	42	52	4.28					
Superior frontal gyrus (BA 6)	−24	42	44	3.27					
Medial frontal gyrus (BA 6)	−34	34	40	3.85					
	−30	36	42	3.65					
	−40	40	26	3.63					
	−32	40	34	3.47					
	−34	42	30	3.45					
	−30	44	32	3.43					
	−40	40	18	3.43					
Orbitofrontal cortex, posterior (BA11)	−28	34	−14	4.08					
Middle frontal gyrus (BA 46)					40	36	30	4.29	830
					42	18	46	4.1	
					38	54	0	3.75	
					40	54	6	3.61	
					46	42	12	3.57	
					40	44	20	3.51	
					30	44	38	3.48	
					42	42	16	3.43	
					38	12	54	3.32	
					36	12	58	3.25	
Superior frontal gyrus (BA 6)					32	60	8	3.42	
Inferior frontal gyrus, pars opercularis (BA 44)					42	16	36	3.54	
					44	16	32	3.45	
					54	16	34	3.36	
Inferior frontal gyrus, pars triangularis (BA 45)					54	22	28	3.49	
					52	26	26	3.44	
Hippocampus					14	−2	−16	4.03	400
Olfactory cortex (BA 25)					8	10	−18	3.48	
					8	16	−14	3.42	
					2	10	−16	3.36	
	−2	10	−12	3.22					
Cerebellum VI					14	12	2	3.82	
Caudate					16	10	12	3.32	
Putamen					26	6	−4	3.56	
					28	4	0	3.48	
					22	6	−6	3.46	
					28	0	8	3.4	
					26	4	14	3.35	
					26	4	6	3.34	
					30	2	4	3.31	
					18	4	−10	3.31	
Putamen	−24	14	−4	3.93					226
	−18	12	−2	3.63					
	−24	2	12	3.55					
	−26	8	8	3.54					
Caudate	−14	12	6	3.17					
Insula	−26	18	−4	3.83					
	−32	10	8	3.34					
	−28	12	8	3.33					
**B. Effect of relief during imagery of tic expression**									
Putamen	−28	−20	−4	4.52					1797
	−24	−20	8	4.09					
	−28	−20	6	4.08					
	−24	10	−2	4					
	−30	8	−2	3.93					
	−22	8	−6	3.87					
	−28	−10	16	3.71					
Insula	−28	−4	18	4.19					
Hippocampus	−30	−8	−12	3.77					
Thalamus (VPL)	−22	−20	4	4.08					
	−16	−18	−2	4.04					
Pallidum	−26	−16	−4	4.39					
	−20	4	−2	3.97					
	−18	4	2	3.81					
	−10	−2	0	3.81					
	−10	−2	−6	3.8					
Pallidum					22	6	2	4.1	238
					18	8	4	4.02	
Putamen					28	2	2	3.99	
					18	12	2	3.7	
Precuneus (BA 30)	−10	−50	8	4.07					1145
	−6	−50	12	4.01					
					4	−50	14	3.89	
	−18	−44	0	3.46					
	−16	−48	2	3.43					
Lingual gyrus (BA 18)	−2	−70	−4	3.78					
Vermis IV−V	0	−60	4	3.62					
Inferior frontal gyrus, pars triangularis	−58	16	8	3.91					437
	−56	20	6	3.77					
	−48	20	16	3.27					
Inferior frontal gyrus, pars opercularis (BA 44)	−44	2	24	3.59					
	−40	4	22	3.39					
	−46	6	28	3.37					
Inferior frontal gyrus, pars orbitalis (BA 38)	−52	22	−4	3.43					
Precentral gyrus (BA 6)	−50	2	20	3.48					
Postcentral gyrus	−58	4	16	3.51					
	−60	0	16	3.28					
Rolandic operculum	−54	2	14	3.74					
	−44	2	18	3.44					
Superior temporal gyrus					52	−16	6	3.5	279
					56	−2	−4	3.47	
					58	−28	8	3.4	
					54	−20	8	3.37	
					56	−24	10	3.34	
Insula					44	2	−2	3.5	
Heschl gyrus					54	−12	6	3.48	
**C. Effect of relief during imagery of tic inhibition**									
Superior frontal gyrus (BA11)					16	56	−8	4.86	61

**A**. Effect of distress during urge imagery. **B**. Effect of relief during imagery of tic expression. **C**. Effect of relief during imagery of tic inhibition. Anatomical labels were taken from the AAL3 template (Brodmann area of the local maxima) and coordinates reported in MNI space. * peak-level *p* < 0.05 FWE, # peak-level *p* < 0.06 FWE.

#### Correlation between level of relief and BOLD signal during the imagery of tic expression

During imagery of tic expression, the activity of different cortical and subcortical regions was positively associated with the perceived relief (Table 2B and Fig. 3B). This network included prefrontal regions, the insular cortex, the superior temporal gyrus, and parieto-occipital regions. We also found clusters of significant activation in the left ventral posterolateral nucleus of the thalamus, and in the dorsal striatum, including the pallidum and the putamen, bilaterally.

#### Correlation between level of relief and BOLD signal during the imagery of tic inhibition

During imagery of tic inhibition, the activity of a cluster encompassing the right superior frontal gyrus and the medial orbitofrontal cortex was negatively associated with the perceived relief (Table 2C and Fig. 3C).

#### Conjunction of the fMRI response for the distress effect during the “tic-inducing scenarios” and relief during “tic imagery”

The significant correlation between the magnitude of the distress felt by the patients when imagining a tic-triggering scenario and the relief they felt when they were allowed to let the imagination of tics go freely (Act phase) justified testing the hypothesis that some brain regions may display a similar effect whereby brain activity was higher the higher the two indexes. The hypothesis was that such brain regions could contribute to urges and tic generation to the same extent, like a spring that accumulates and then releases energy (spring model of tic urge and generation). We found one region that satisfied such hypothesis: the left putamen where a conjunction of the two linear regressions was found (stereotactic coordinates: −24 14 −4; Z-score: 3.8; *p* < 0.00005; cluster level significance: *p* < 0.05 uncorrected, see Fig. S2).

#### Shared neural substrates of tic behavior and tic imagery

These results are described in the supplementary materials (Fig. S3).

## Discussion

We previously provided evidence of a different neurofunctional organization of motor control in GTS, entailing different phases of motor execution (i.e., motor planning, motor execution, and motor awareness [25–27]). Here, we expand such findings demonstrating that the neural patterns observed are even more marked when considering tic-related phenomenology rather than ordinary motor acts, especially if one considers the specific sensations perceived by GTS patients during the execution of a ticking-related task.

We created a novel experimental paradigm based on mental imagery to study the neurofunctional correlates of distress and relief associated with tic behaviors in GTS patients. Our patients were instructed to mentally recall typical tic-triggering scenarios, followed by the simulation of tic behaviors or their inhibition. We compared the brain activation patterns evoked by these scenarios with the neural activity recorded during the mental simulation of “neutral situations” that were then followed by the mental execution or inhibition of pre-determined stereotyped motor behaviors.

We measured the distress, or the relief experienced during the different phases of the tasks, by using the mean of subjective trial-by-trial reports. This allowed us to infer that this mental simulation effectively evoked tic-related scenarios (e.g., a greater level of distress perceived during the imagery of tic-related scenarios) and to test whether the mental generation of tics produced a sort of temporary relief from this distress. Crucially, we correlated these self-reports with the neural activity recorded during each task phase.

Before further do, we can comment on the fact that some of the hypotheses made in the introduction are not confirmed by the data: for example, the idea that tics may represent some form of pathologically motivated behavior. We did not observe signals in regions like the ventral tegmental area or the nucleus accumbens that are traditionally associated with motivated behaviors and rewards like, for example, food or drugs. In other words, no simple metaphor of tics and the associated feelings is supported by our data, at least not in a generic manner.

In the following paragraphs, all considerations are made by taking for granted that the effects commented upon (behavioral and fMRI) were over and above what was measured in the control conditions.

### Behavioral findings

Self-reports collected throughout the different phases of the tasks revealed that the distress perceived during the imagination of tic-triggering scenarios was significant, validating our experimental design and indicating that contextual factors significantly influence tic manifestations, even when mentally recalled rather than experienced in daily life.

Self-report data also showed that the simple imagination of tics could provide relief from the distress perceived during the preceding “evoking” phase. Notably, tic imagery did not cause an increase of tic-like actual movements: head movements recorded during the MRI scanning did not significantly increase during tic imagery. This relief, as expected, was not present when subjects were instructed to imagine tic inhibition, confirming the specific “relieving” function of tic imagination.

### Neural correlates of mental imagery

At the neurofunctional level, our mental imagery task – across conditions and phases – was associated with the activation of a large frontoparietal network comprising prefrontal, premotor and somatosensory regions, showing that several cortical areas playing a role during actual behaviors are also consistently activated during imagery. As expected, we observed that this neural network was more active in the “*act*” and “*inhibit”* phases than in the “*urge to tic - triggering context recall*” one. Crucially, the mental imagery network was similarly recruited in the tic and in the control scenarios, in line with the self-reports on the quality of participants’ imagery: the Visual Analogue Scales data were similar for the two scenarios.

These findings, combined with our behavioral evidence indicating good imagery abilities in our participants, favor the idea that our participants could perform a mental imagery task and were strongly engaged in the experiment.

### Neural correlates of tic urges and tic imagery, their association with distress or relief

Our investigation started with considerations on what are the distinctive features of tics (urges, generation, or inhibition) and how we can distinguish them from other motor activities. Of course, the level of voluntariness is one distinctive trait: tics are somewhere in between fully voluntary and involuntary acts, yet one could argue that it is impossible to find a reference task for tics during an imaging study because no action can be generated, even a conditional one, without some degree of voluntariness. Here, to better characterize the brain physiology of tics, we exploited one distinctive aspect of the tic-related phenomenology: the patients’ subjective and affective experience. It is telling that most of the fMRI findings were due to the correlations of the BOLD response with the level of distress or relief experienced by the participants in different phases of the tasks. Importantly, because the level of distress and relief experienced during the “urge phase” and “act phase” respectively, were correlated, we were able to test the hypothesis that the activity of some brain regions was equally relevant for these two moments in the manifestation of the tic-related subjective feelings. One or more such regions could be a good candidate for an overall causal role in tic-related phenomenology.

### Brain activity during the retrieval of scenarios associated with urge to tic

The simple imagination of a scenario, that favors the occurrence of tics and the associated uncomfortable sensation of distress typically present before tic generation, was associated with a bilateral brain network including lateral and mesial premotor regions, and parietal regions (somatosensory cortices and the parietal operculum).

These findings are consistent with previous fMRI studies [18, 19], where a similar activation pattern was recorded before actual tic manifestations. Coherently, it has been shown that the electrical stimulation of SMA triggers subjective-sensory responses like an urge to perform a movement or the anticipation that a movement is about to occur [28]. Notably, the SMA is one of the mesocortical target of dopaminergic projections that arise from mid-brain structures, such as substantia nigra and ventral tegmental area [29, 30]. The involvement of these systems (during the tic-evoking phase) and basal ganglia (during both urges for tics and tic manifestations, see below) is coherent with the efficacy of anti-dopaminergic treatment on tics in GTS patients [31]. Hence, clinical, anatomical, and neurochemical data suggest that excessive fronto-mesocortical discharges may facilitate tics in GTS. Our findings and previous studies highlighting abnormal SMA activation in GTS patients support this view [14, 25–27].

This sensorimotor cortical network activated during the mental evoking of tic-triggering scenarios may represent the neural substrate for the uncomfortable feelings associated with premonitory urges of tics, even during their simple imagination. It remains to be discussed to what extent these networks are causally related to tic urges and generation.

One possibility could be that the premotor and sensory-motor patterns, and their correlation with distress, might simply represent the equivalent of a motoric pre-alarm as the one demonstrated in motor readiness paradigms also in healthy subjects [32], rather than a brain pattern causally related to GTS and its tics. This is something that we shall discuss below further.

### Brain activity for tic imagery execution/inhibition and subjective relief

The correlation of the tic imagery condition with the level of relief experienced by the participant identified specific brain patterns mainly including subcortical regions rather than the cortical ones, comprising the motoric/somatosensory thalamic nuclei, the pallidum, and the putamen.

While the precise mechanisms underlying the role of the thalamus and basal ganglia in GTS are still not fully understood, evidence from deep brain stimulation studies suggests that dysfunction in these brain regions may play a significant causal role in the disorder (review in ref. [33]).

Combining these neurofunctional results with the behavioral evidence of distress reduction during tic imagery, we can hypothesize that tic imagery might involve the same neural pathways that control actual movement. When patients imagine performing a tic, similar neural pathways are activated but without actual tic expression, reducing tic-related distress.

Finally, a lower relief during tic inhibition was associated with greater (the need of) activation of the orbitofrontal cortex (OFC). Our data support the idea that the more this region was active the more tic inhibition was associated with personal distress. A failure in this mechanism might contribute to the unavoidable manifestation of tics. GTS has been primarily related to dysfunction of the sensorimotor pathways, but recent studies in pediatric GTS patients highlighted abnormal OFC white matter [34, 35]. Further, OFC is implicated in the control of impulsive behaviors [36, 37], and it showed higher activity in trials in which there was a conflict between goal-directed and habitual responses [38]. We propose that the activity of the OFC might serve as a top-down control over involuntary behaviors, such as tics, during their urges and even more so during their voluntary inhibition.

### The conjunction of the effects of distress during urges and relief during tic imagery

The magnitude of the distress and relief during the two phases of the protocol did correlate significantly at the behavioral level permitting to formulate the hypothesis that any brain region jointly significant in its correlation with the BOLD signal of the two phases could have a role of special importance in GTS: when tested with the most stringent form of conjunction analysis permitted by SPM, one such region was found, in the left putamen. The putamen is a well-known part of the motor circuitry, and its dorsal subdivision contributes with its plastic changes to the consolidation of (motor) habits [39]. A mechanistic functional interpretation of this finding remains, of course, still speculative and in need of more direct evidence, perhaps using DBS recordings preceding and during tics. One possible interpretation that we offer here is that, at variance of other brain regions that contribute to specific aspects of the physiology described, the putamen may be the main source of the tic phenomenology: its activation pattern scales not only with the distress perceived during urges but also with the relief during tic imagery. This pattern is reminiscent of what a springboard would normally do when accumulating - distress during urges - and discharging energy with its associated relief during tic imagination.

### Clinical considerations

Our results emphasize the importance of exploring the relationship between the physiology of the tic-related phenomenology and personal distress or relief experienced by patients. We propose that the distress experienced during urges might be the causing factor of the activations seen in cortical premotor networks. Moreover, as the relief during tic imagery was higher the higher the subcortical structures activation, these structures may play a causal role in the affective component of tic phenomenology. Having shown that at least one brain region is associated with both the struggle due to tic urges and the relief experienced during tic imagery justifies some clinical considerations.

The evidence that tic mental imagery can modulate patients’ clinical manifestations and distress suggests that this non-invasive and safe behavioral technique could be used in conjunction with other treatments, such as medication and behavioral therapies. Patients could use tic imagery when they feel the urge to perform a tic to dissipate the distress associated with urges.

More specifically, tic imagery might be integrated with Habit Reversal Training, a behavioral therapy technique commonly used to manage tics associated with GTS. Our evidence suggests that this technique could be complemented with tic imagery, given its relief effect on GTS patients.

Of course, this suggestion will require a proper clinical trial for a convincing demonstration: yet our neurofunctional and behavioral data represent a proof of concept in this direction.

### Limitations

There are some limitations in our approach that we wish to acknowledge. We are aware that the co-occurrence of medication hampers any firm conclusion based on imaging findings about the true nature of GTS but makes the observations relevant for a substantial proportion of adult GTS patients from the real world, namely medicated patients. Observations in un-medicated GTS patients would allow one to describe GTS as it develops naturally in adulthood. Such patients would perhaps be from a milder part of the GTS spectrum, making the observations in such samples non-generalizable. We believe that our sample is representative of what clinicians see in their daily practice with adult GTS patients when the symptoms are clinically relevant.

We also acknowledge cross-sectional nature of our study without a control group could be seen as a limitation. However, we believe that the formal comparison of our experimental task with a perfectly matched baseline condition allowed us to “subtract” the neural activity generally associated with motor behavior not directly related to the tic dimension.

### Supplementary information

Supplementary Materials

## Data Availability

All data, code, and materials are available upon request to the corresponding author LZ.

## Material availability

All data, code, and materials are available upon request to the corresponding author LZ.

## Code availability

All data, code, and materials are available upon request to the corresponding author LZ.
