# Supplementary material for: Mapping Gilles de la Tourette syndrome through the distress and relief associated with tic-related behaviors: an fMRI study
Source: Transl Psychiatry. 2024 Jan 8;14:7. doi: 10.1038/s41398-023-02711-z (PMC10774308; doi:10.1038/s41398-023-02711-z)
Supplement: Supplementary file 1 — Supplementary Materials [file 41398_2023_2711_MOESM1_ESM.pdf]

Supplementary Materials

Mapping Gilles de la Tourette Syndrome through the distress and relief associated with tic-related behaviors: an fMRI study

Laura Zapparoli[1,2*#], Francantonio Devoto[1#], Marika Mariano[1], Silvia Seghezzi[1,3], Domenico Servello[4], Mauro Porta[4] & Eraldo Paulesu[1,2*]

[1] Psychology Department and NeuroMi – Milan Centre for Neuroscience, University of Milano-Bicocca, Milan, Italy

[2] fMRI Unit, IRCCS Orthopedic Institute Galeazzi, Milan, Italy

[3]Institute of Cognitive Neuroscience, University College London, London, UK

[4] Tourette Center, IRCCS Orthopedic Institute Galeazzi, Milan, Italy

***Corresponding authors**

Laura Zapparoli and Eraldo Paulesu

Psychology Department

University of Milano-Bicocca

Email: laura.zapparoli@unimib.it, eraldo.paulesu@unimib.it

# These authors equally contributed to the paper and shared first authorship.

Running title: Brain correlates of tic-related behaviors

Materials and Methods

Sample size calculation

In order to determine the sample size of the study, we carried out an a-priori power analysis on the basis of the scientific literature. We used as a reference a published study addressing the changes in tic-related behaviors in two different observational settings (Piacentini et al., 2006[1]). In particular, we observed that the effect size of this difference was 0.62. On the basis of these data, we calculated that the selection of a sample of 23 participants would allow us to detect a significant difference between the two conditions in terms of premonitory urges, with a power of 0.8 and an alpha of 0.05. On the basis of this analysis, we recruited 25 participants (to take into account the possible dropouts).

Neuropsychological and psychopathological assessment

The neuropsychological screening included the Mini-Mental State Examination (MMSE[2]), the Raven's Coloured Progressive Matrices (Raven's Matrices[3]) and the Frontal Assessment Battery (FAB[4]). None of the subjects had pathological scores at any of the aforementioned tests. The psychopathological test battery included: the Barratt Impulsivity Scale (BIS) for impulsivity[5]; the Yale-Brown Obsessive Compulsive Scale for obsessive-compulsive disorder (YBOCS[6]), the Beck Depression Inventory for depression assessment (BDI[7]) and the Adult ADHD Self-Report Scale for the attention deficit hyperactivity disorder (ASRS[8]). Moreover, patients underwent a detailed interview about the severity of their motor symptoms, including the Yale Global Tic Severity Scale (YGTSS[9]) and the Premonitory Urge Tics Scale (PUTS[10]).

Data acquisition

MRI scans were performed using a 1.5 T Siemens *Avanto* scanner, equipped with gradient-echo echo-planar imaging (flip angle 90°, TE=40 msec, TR=2000 msec, FOV=250 mm and matrix=64x64). The overall number of fMRI volumes collected was 400 per subject. The first 15 volumes of each sequence (corresponding to the presentation of the task instructions) were discarded from the analyses.

Pre-processing

After the image reconstruction, raw data visualization and conversion from DICOM to the NIFTI format were performed with MRIcron (www.mricro.com) software. All subsequent data analyses were performed in MATLAB R2019b (Mathworks. Natick. MA. USA) using the software Statistical Parametric Mapping (SPM12, Wellcome Department of Imaging Neuroscience, London, UK). First, fMRI scans were realigned to the first image of the run to account for any movement during the experiment. The realigned images were stereotactically normalised into the MNI-EPI fMRI template space to permit group analyses of the data[11,12]. At this stage, the data matrix was interpolated to produce voxels with the dimensions 2x2x2 mm. The stereotactically normalized scans were smoothed using a Gaussian filter of 10 x 10 x 10 mm to improve the signal-to-noise ratio, making the data suited for cluster-level correction for multiple comparisons[13].

Moreover, we used Artifact detection Tools to exclude fMRI scans contaminated by tics (Withfield-Gabrieli, https://www.nitrc.org/projects/artifact_detect/). This toolbox allows identifying and discarding from the analyses the scans that could lead to artefactual statistical effects due to excessive movement. Thresholds were set at 2 mm scan-to-scan head movement and 9 standard deviations of scan-to-scan global signal intensity change. Experimental subjects exhibiting more than 20% outlier scans in the whole experimental run were to be excluded from the subsequent statistical analyses.

None of the participants included in the final sample exceeded this threshold (M = 3.93 %, SD = 4.97 % scan outliers). Furthermore, the number of scan outliers between the tic and the control condition was not significantly different (paired samples Wilcoxon test; V = 36, p = .85).

First-level fixed-effect analyses
The BOLD signal associated with each experimental condition was analyzed by a convolution with a canonical hemodynamic response function[14]. Global differences in the fMRI signal were removed from all voxels with proportional scaling. High-pass filtering (128 s) was used to remove artefactual contributions to the fMRI signal, such as physiological noise from cardiac or respiratory cycles. The six motion parameters generated by the realignment procedure and the regressors generated by the Artifact Detection Tools toolbox were added to the design matrix in the first-level analyses to partial-out the impact of motion artifacts on the estimates of the beta parameters.

Correlations with demographic and clinical data.
We performed additional analyses to regress out the effect of demographic variables (age, gender) and symptom severity (YGTSS total score) from the relationship between BOLD activity and tic-related phenomenology.
For each scenario (urge, tic, inhibition), we performed three second-level analyses where age, gender, and YGTSS scores were inserted as covariates in the model. For each model, we computed the following contrasts: (i) effect of interest (testing the relationship between BOLD and distress/relief after controlling for age, gender, or YGTSS); (ii) effect of nuisance covariate (relationship between BOLD activity and age, gender, or YGTSS).
No significant association was found between the BOLD activity and age, gender, or YGTSS total score, making it unlikely that sociodemographic variables and symptom severity affected our results.

Correlations with neuroleptic medication levels
To investigate whether neurofunctional results were influenced by the neuroleptic medication levels (given their effect on the motor system even at low doses), we performed a series of correlations between the BOLD signal collected during the different conditions of the experiment and chlorpromazine equivalent scores (https://cpnp.org/guideline/essentials/antipsychotic-dose-equivalents). None of the analyses highlighted a significant association between the BOLD activity and the medication dosage.

Behavioral results
During the *urge* phase, subjects reported higher *perceived distress* in the tic (M = 3.78, SD = 1.08) compared with the control scenario (M = 2.02, SD = 1.05); t(22) = 4.51, $p < .001$ (Figure 2A, top left). During the *behavior act* phase, subjects reported higher *perceived relief* in the tic (M = 3.77, SD = 0.91) compared with the control condition (M = 2.95, SD = 1.31); t(22) = 2.18, p = .04 (Figure 2A, top right), whereas during the *behavior inhibit* scenario they did not report different ratings between the tic (M = 3.30, SD = 1.09) and the control (M = 3.33, SD = 1.37) condition (t(21) = -0.09, p = .93) (Figure 2A, bottom left).
After fMRI, the average report of the *quality of the imagery* between the experimental tic task (M = 7.41, SD = 1.8) and the control task (M = 7.18, SD = 1.76) was not significantly different (t(22) = 0.46, p = .65), indicating similar imagery abilities in both scenarios (Figure 2A, bottom right).

fMRI Results

Main effect of motor imagery

The results showed large clusters of significant bilateral activations in the prefrontal cortex, including the SMA, the middle cingulum, the middle and inferior frontal gyri (IFG pars triangularis and opercularis), the inferior parietal cortex and the angular gyrus, and the right insula (Table S2A and Figure S1A).

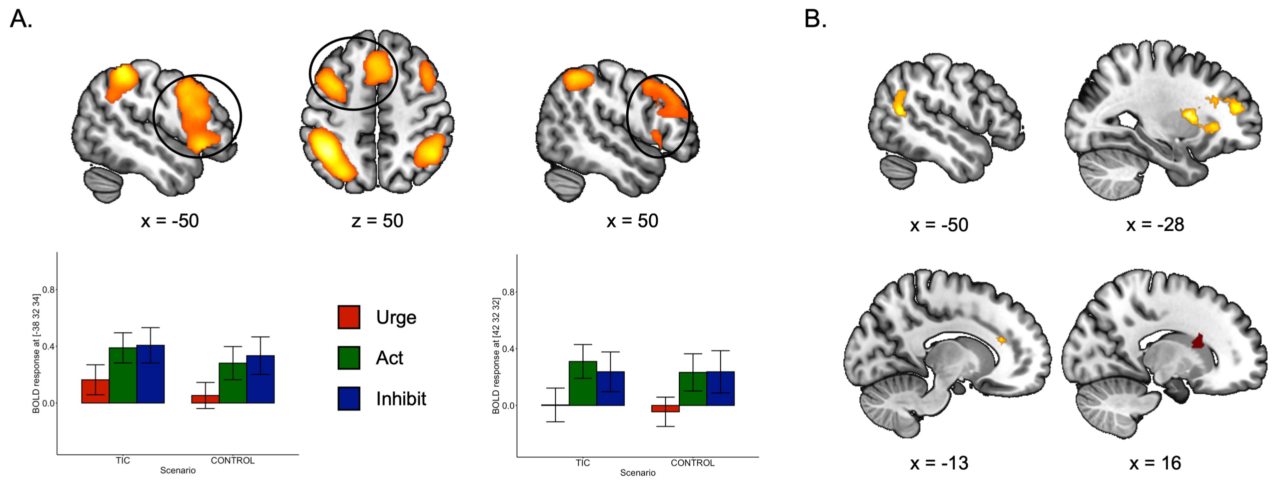

Figure S1. A. Main effect of motor imagery (Behavior Phase > Urge Phase) for the experimental and control tasks. **B.** Main effect of urge phase.

Urge phase (Tic scenario > Control scenario)

The results showed a left-lateralized frontal-parietal network comprising the superior and middle frontal cortex, the inferior frontal gyrus, the superior part of the anterior cingulate cortex, the anterior insula, the inferior parietal cortex, including the supramarginal and angular gyri, and the middle temporal gyrus. We also observed a significant peak of activity in the right caudate nucleus surviving voxel-level FWE correction ($p < .01$, Table S2B and Figure S1B).

Behavior phase (Act/Inhibit Tic scenario > Control scenario).

No voxel survived the predefined statistical thresholds.

Conjunction of the fMRI response for the distress effect during the "Urge Phase" and relief during "Tic imagery Phase".

The significant correlation between the magnitude of the distress felt by the patients when imagining a tic-triggering scenario and the relief they felt when they were allowed to let the imagination of tics go freely (Act phase) justified testing the hypothesis that some brain regions may display a similar effect whereby brain activity was higher the higher the two indexes. The hypothesis was that such brain regions could contribute to urges and tic generation to the same extent, like a spring that accumulates and then releases energy (spring model of tic urge and generation). We found one region that satisfied such hypothesis: the left putamen where a conjunction of the two linear regressions was found (stereotactic coordinates: -24 14 -4; Z-score: 3.8; $p < 0.00005$; cluster level significance: $p < 0.05$ uncorrected, see Figure S2).

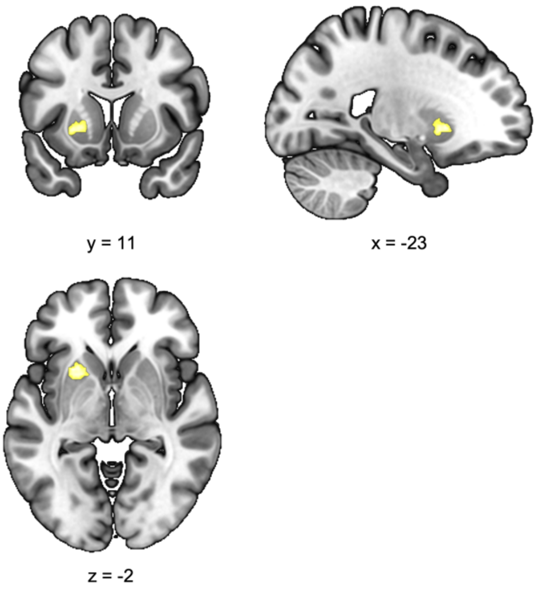

Figure S2. The conjunction of the fMRI response for the distress effect during the “Urge Phase” and relief effect during “Tic imagery execution Phase”.

Shared neural substrates of tic behavior and tic imagery

To provide more direct experimental evidence on the shared neural activity between actual and imagined tic behavior, we performed a meta-analysis of neuroimaging studies on the neural bases of tic generation and suppression in GTS patients. Stereotactic coordinates reported by previous studies on tic generation and suppression[15-18] were submitted to the Activation Likely Estimation (ALE) algorithm implemented in the GingerALE software. In brief, the procedure returns a map representing the brain regions that show significant anatomofunctional convergence across studies ($p < .001$, uncorrected).

The meta-analytical activation map of tic generation (Figure S3, red) and the T-map of the neural network associated with tic imagery (Figure S3, green) were overlaid on an anatomical template. The two maps intersect bilaterally in the insula and in the basal ganglia, including pallidum and putamen, suggesting a partial overlap between the brain network involved in actual tic behavior and the brain areas involved in tic imagery (Figure S3, yellow).

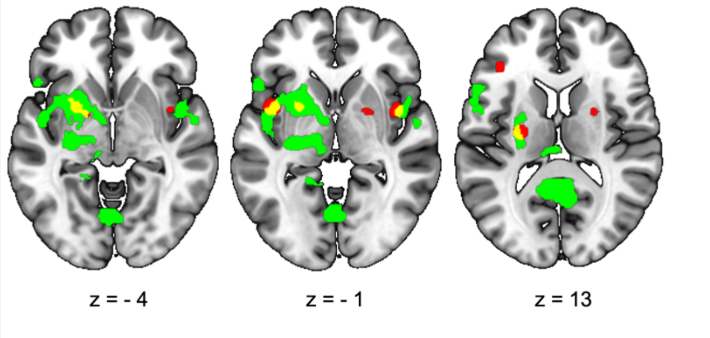

Brain Area	Overlap with fMRI Tic map (%)
Rolandic_Oper_L 2331	11.9
Insula_L 3001	18.8
Insula_R 3002	14.6
Putamen_L 7011	66.6
Pallidum_L 7021	81.4
Thalamus_L 7101	18.1
Temporal_Sup_L 8111	2.1

Figure S3. Shared neural substrates of actual and imagined tic behavior. Left: axial slices showing the brain regions associated with tic generation and suppression (meta-analytical results, red), the brain regions associated with tic imagery (present study results, green), and their overlap (yellow). Activation maps were overlaid on an anatomical template in MNI space. Right: table reporting the proportion of overlapping voxels between the meta-analytic map and the activation map for the tic imagery scenario.

Supplementary references

1 Piacentini, J. *et al.* Reactivity of tic observation procedures to situation and setting. *J Abnorm Child Psychol* **34**, 649-658 (2006). https://doi.org/10.1007/s10802-006-9048-5

2 Folstein, M. F., Folstein, S. E. & McHugh, P. R. "Mini-mental state". A practical method for grading the cognitive state of patients for the clinician. *J Psychiatr Res* **12**, 189-198 (1975). https://doi.org/10.1016/0022-3956(75)90026-6

3 Raven, J., Raven, J. & Court, J. *Manual for Raven's Progressive Matrices and Vocabulary Scales*. (1998).

4 Dubois, B., Slachevsky, A., Litvan, I. & Pillon, B. The FAB: a Frontal Assessment Battery at bedside. *Neurology* **55**, 1621-1626 (2000). https://doi.org/10.1212/wnl.55.11.1621

5 Fossati, A., Di Ceglie, A., Acquarini, E. & Barratt, E. S. Psychometric properties of an Italian version of the Barratt Impulsiveness Scale-11 (BIS-11) in nonclinical subjects. *J Clin Psychol* **57**, 815-828 (2001). https://doi.org/10.1002/jclp.1051

6 Goodman, W. K. *et al.* The Yale-Brown Obsessive Compulsive Scale. I. Development, use, and reliability. *Arch Gen Psychiatry* **46**, 1006-1011 (1989). https://doi.org/10.1001/archpsyc.1989.01810110048007

7 Beck, A. T. An Inventory for Measuring Depression. *Arch Gen Psychiatry* **4**, 561-571 (1961).

8 Adler, L. A. *et al.* Validity of pilot Adult ADHD Self- Report Scale (ASRS) to Rate Adult ADHD symptoms. *Ann Clin Psychiatry* **18**, 145-148 (2006). https://doi.org/10.1080/10401230600801077

9 Leckman, J. F. *et al.* The Yale Global Tic Severity Scale: initial testing of a clinician-rated scale of tic severity. *J Am Acad Child Adolesc Psychiatry* **28**, 566-573 (1989). https://doi.org/10.1097/00004583-198907000-00015

10 Woods, D. W., Piacentini, J., Himle, M. B. & Chang, S. Premonitory Urge for Tics Scale (PUTS): initial psychometric results and examination of the premonitory urge phenomenon in youths with Tic disorders. *J Dev Behav Pediatr* **26**, 397-403 (2005). https://doi.org/10.1097/00004703-200512000-00001

11 Ashburner, J. & Friston, K. Nonlinear spatial normalization using basis functions. *Human Brain Mapping* **7**, 254-266 (1999).

12 Friston, K. J. *et al.* Analysis of fMRI time-series revisited. *Neuroimage* **2**, 45-53 (1995). https://doi.org/S1053-8119(85)71007-510.1006/nimg.1995.1007

13 Flandin, G. & Friston, K. J. Analysis of family-wise error rates in statistical parametric mapping using random field theory. H*um Brain Mapp* 4**0,** 2052-2054 (2019). https://doi.org/10.1002/hbm.23839

14 Worsley, K. J. & Friston, K. J. Analysis of fMRI time-series revisited--again. N*euroimage* 2**,** 173-181 (1995). https://doi.org/10.1006/nimg.1995.1023

15 Ganos, C. *et al.* Prefrontal cortex volume reductions and tic inhibition are unrelated in uncomplicated GTS adults. J *Psychosom Res* 7**6,** 84-87 (2014). https://doi.org/10.1016/j.jpsychores.2013.10.014

16 Stern, E. *et al.* A functional neuroanatomy of tics in Tourette syndrome. A*rch Gen Psychiatry* 5**7,** 741-748 (2000). https://doi.org/yoa8250 [pii]

17 Lerner, A. *et al.* Neuroimaging of neuronal circuits involved in tic generation in patients with Tourette syndrome. N*eurology* 6**8,** 1979-1987 (2007). https://doi.org/10.1212/01.wnl.0000264417.18604.12

18 Bohlhalter, S. *et al.* Neural correlates of tic generation in Tourette syndrome: an event-related functional MRI study. B*rain* 1**29,** 2029-2037 (2006). https://doi.org/awl050 10.1093/brain/awl050

Table S1. Current motoric and sound tics at the moment of the experiment, as assessed with the YGTSS.

# Patient	YGTSS – Motor tics	YGTSS – Sound Tics
1	Eye blinking, eye movements, head movements, arm movements, hand movement, leg movements	Coughing, throat clearing, sniffing
2	Back bending	Complex phonic tic
3	Shoulder movements, facial expressions (surprise), head movements, mouth movements, back bending, head gestures (nodding)	Throat clearing
4	Eye movements, head movements, arm movements, hand movements	Sniffing
5	Eye movements, eye blinking, mouth movements, leg movements, feet movements, toe movements, dystonic postures, arm movements	Coughing
6	Eye movements, eye blinking, head movements, arm movements, hand movements, nails biting, fingers cracking, leg movements, feet movements	Throat clearing

7	Eye movements, eye blinking, mouth movements, shoulder movements, hand movements, leg movements, feet movements	Coughing, throat clearing, pronouncing "m" sound
8	Eye movements, eye blinking, head movements, shoulder movements, arm movements, hand movements, nails biting, fingers cracking, leg movements, feet movements	Swallowing
9	Eye movements, eye blinking, nose movements, tongue biting, fingers cracking, leg movements, feet movements, sniffing objects	--
10	Eye movements, eye blinking, head gestures (nodding), touching hairs, hand movements, leg movements, feet movements, back cracking, dystonic postures	Guttural sound, rapid changes of speech tone or volume
11	Eye movements, eye blinking, nose movements, tongue biting, lips biting and licking, mouth movements, head movements, shoulder movements, leg movements, feet movements	Throat clearing, swallowing, sniffing, echolalia (sounds, words, sentences)
12	Eye movements, eye blinking, shoulders movements, toe movements, head scratching	--
13	Eye movements, eye blinking, shoulders movements, toe movements	Animal or bird noises

14	Nose movements, head movements, shoulder movements, arm movements, hand movements, leg movements	Coughing, throat clearing, sniffing, whistling
15	Eye movements, eye blinking, facial expressions (surprise, smili, funny expressions), nose movements, head movements, tongue and lips biting, mouth movements, shoulder movements, arm movements, hand movements, touching, tapping, grabbing objects, risky behaviors, copropraxia, dystonic postures, leg movements, feet movements, bruxism	Throat clearing, syllabes, rapid changes of speech tone and volume
16	Eye movements, eye blinking, nose movements, head movements, tongue biting, lips licking, shoulder movements, fingers cracking, arm movements, hand movements, leg movements, touching objects, tapping, dystonic postures, tricotillomania	Throat clearing, moaning
17	Eye movements, eye blinking, facial expressions (surprise), head movements, dystonic postures, back bending	Coughing
18	--	--
19	Eye movements, eye blinking, lips licking, nose movements, shoulder movements, nails biting, fingers cracking, leg movements, feet movements	Sniffing

20	Nose movements, mouth movements, facial expressions, arm movements, hand movements	Throat clearing, swallowing, sniffing
21	Nose movements, mouth movements, toe movements, echopraxia	Throat clearing, whistling, rapid changes of speech tone and volume
22	Eye movements, eye blinking, facial expressions (surprise), mouth movements, shoulder movements, head movements, arm movements, hand movements, leg movements, feet movements	Throat clearing, whistling, rapid changes of speech tone and volume
23	Eye movements, eye blinking, facial expressions, mouth movements, shoulder movements, head movements, arm movements, hand movements, leg movements, feet movements, touching objects	Throat clearing, coughing, syllabes
24	Eye movements, eye blinking, lips licking, nose movements, lips biting, shoulder movements, head movements, arm movements, hand and finger movements, hair touching,	Coughing, throat clearing, blowing
25	Shoulder movements, arm movements, hand movements, leg movements, feet movements, toe movements	Coughing, syllables, coprolalia

Table S2. fMRI results of the full factorial and one sample t-test. A. Main effect of task. **B.** Main effect of urge. Anatomical labels were taken from the AAL3 template (Brodmann area of the local maxima) and coordinates reported in MNI space. * peak-level p < .05 FWE, ** peak-level p < .01 FWE, # peak-level p < 0.06 FWE.

	Left hemisphere				Right hemisphere				
**Anatomical label (Brodmann area)**	**x**	**y**	**z**	**Z-value**	**x**	**y**	**z**	**Z-value**	**Cluster size (voxels)**
**A. Main effect of task**									
Inferior parietal lobule (BA 40)	-42	-48	56	5.97*					3031
Superior parietal lobule (BA 7)	-30	-66	54	4.85*					
Middle frontal gyrus (BA 45)					42	32	32	4.7*	2738
					40	36	30	4.67*	
					40	44	20	4.37	
					42	24	42	4.31	
					44	12	48	4.03	
					40	12	50	3.98	
					34	-2	60	3.23	
Superior frontal gyrus (BA 46)					34	54	20	4.11	
Inferior frontal gyrus, pars opercularis (BA 44)					58	16	2	3.66	
					56	18	18	3.38	
					52	14	34	3.19	
Inferior frontal gyrus, pars triangularis (BA 45)					54	22	24	3.38	
Precentral gyrus (BA 6)					48	8	46	3.97	
Middle frontal gyrus (BA 45)	-38	32	34	4.69*					6545
	-34	4	52	4.56*					
	-38	44	8	4.26					
	-34	46	14	4.25					
	-42	24	34	4.47#					

Inferior frontal gyrus, pars triangularis (BA 45)	-48	20	22	4.15					
	-40	32	8	4.06					
	-46	28	22	3.97					
	-40	36	24	3.96					
Inferior frontal gyrus, pars orbitalis (BA 38)	-50	22	-6	4.51*					
Supplementary motor area (BA 6)	0	12	56	4.49#					
	-10	12	64	4.09					
	-2	22	52	4.31					
Precentral gyrus (BA 6)	-44	10	48	4.41					
	-46	8	44	4.4					
	-48	12	32	4.29					
Inferior parietal lobule (BA 40)					46	-52	50	4.68*	1075
**B. Main effect of urge**									
Superior anterior cingulate cortex (BA 32)	-18	30	20	4.69#					593
Middle frontal gyrus (BA 45)	-38	42	16	4.18					
	-32	48	18	3.99					
	-22	48	14	3.97					
	-28	46	16	3.81					
	-24	30	24	3.41					
	-26	34	24	3.28					
	-28	38	26	3.26					
Superior frontal gyrus (BA 46)	-28	44	20	3.72					
Inferior frontal gyrus, pars trinagularis (BA 45)	-30	22	26	3.83					
	-44	36	6	3.52					
Insula	-22	30	10	3.25					
Middle temporal gyrus (BA 37)	-48	-58	14	4.66					269

Angular gyrus (BA 39)	-54	-58	28	3.44					
	-46	-58	32	3.32					
Insula	-32	12	4	4.05					
	-34	22	2	4.04					
	-34	8	4	3.9					
	-36	18	-2	3.67					
	-34	16	6	3.66					
Putamen	-28	4	12	4.4					494
	-28	8	8	4.22					
	-28	-2	16	3.46					
Caudate					20	14	18	5.26**	213
**C. Behavior (act and inhibit) > Context**									
Supplementary motor area (BA 6)	0	8	58	6.34*					1323
Middle cingulate gyrus (BA 32)					12	14	44	3.23	
					12	16	40	3.23	
Inferior frontal gyrus, pars triangularis (BA 47)	-46	28	0	5.97*					5952
	-48	32	-4	5.95*					
	-36	26	30	5.65*					
	-40	30	8	5.28*					
	-36	28	10	4.99*					
	-42	16	30	4.69*					
	-46	20	28	4.65*					
	-32	30	14	4.63*					
Inferior frontal gyrus, pars opercularis (BA 44)	-50	14	2	5.38*					
Middle frontal gyrus (BA 45)	-38	30	32	5.42*					
	-38	4	56	5.3*					
	-28	42	20	5.17*					

	-34	38	22	5.05*					
	-38	34	22	4.82*					
	-34	34	16	4.43					
Inferior parietal lobule (BA 40)	-50	-46	54	4.49#					1899
	-42	-56	50	4.37					
	-46	-52	52	4.3					
	-42	-54	42	4.16					
	-54	-44	36	4.01					
Angular gyrus (BA 39)	-50	-68	28	3.15					
	-56	-66	24	3.12					
	-40	-58	34	4.38					
	-44	-56	38	4.22					
	-44	-60	40	4.05					
Inferior parietal lobule (BA 40)	-34	-70	50	3.61					
Supramarginal gyrus (BA 40)	-52	-50	34	3.72					
Insula					46	12	0	4.24	407
					38	18	4	3.7	
					34	22	8	3.54	
